# Supplementary material for: A social ecological approach to identify the barriers and facilitators to COVID-19 vaccination acceptance: A scoping review
Source: PLoS One. 2022 Oct 3;17(10):e0272642. doi: 10.1371/journal.pone.0272642 (PMC9529136; doi:10.1371/journal.pone.0272642)
Supplement: S1 Table — (DOCX) [file pone.0272642.s001.docx]

**S1 Table: Databases search strategy**

**Full search strategy for PubMed:**

| **Search number** | **Query** | **Search Details** | **Results** |
| --- | --- | --- | --- |
| 11 | #7 AND #10 | ("vaccines"[MeSH Terms:noexp] OR ("vaccine"[Title/Abstract] OR "vaccines"[Title/Abstract] OR "vaccination"[Title/Abstract] OR "vaccinations"[Title/Abstract])) AND ("COVID-19"[MeSH Terms:noexp] OR ("COVID-19"[Title/Abstract] OR "ncov*"[Title/Abstract] OR "2019nCoV"[Title/Abstract] OR "19nCoV"[Title/Abstract] OR "covid19*"[Title/Abstract] OR "COVID"[Title/Abstract] OR "SARS-COV-2"[Title/Abstract] OR "SARSCOV-2"[Title/Abstract] OR "SARSCOV2"[Title/Abstract] OR "Severe Acute Respiratory Syndrome Coronavirus 2"[Title/Abstract] OR "Severe Acute Respiratory Syndrome Corona Virus 2"[Title/Abstract])) AND ("vaccination refusal"[MeSH Terms:noexp] OR "anti vaccination movement"[MeSH Terms:noexp] OR "mass vaccination"[MeSH Terms:noexp] OR "vaccination coverage"[MeSH Terms:noexp] OR ("hesitan*"[Title/Abstract] OR "acceptance"[Title/Abstract] OR "preference"[Title/Abstract] OR "rejection"[Title/Abstract] OR "anti vaccin*"[Title/Abstract] OR "attitude"[Title/Abstract] OR "attitudes"[Title/Abstract] OR "barrier"[Title/Abstract] OR "barriers"[Title/Abstract] OR "facilitator"[Title/Abstract] OR "facilitators"[Title/Abstract] OR "intent*"[Title/Abstract])) | 786 |
| 10 | #8 OR #9 | "vaccination refusal"[MeSH Terms:noexp] OR "anti vaccination movement"[MeSH Terms:noexp] OR "mass vaccination"[MeSH Terms:noexp] OR "vaccination coverage"[MeSH Terms:noexp] OR "hesitan*"[Title/Abstract] OR "acceptance"[Title/Abstract] OR "preference"[Title/Abstract] OR "rejection"[Title/Abstract] OR "anti vaccin*"[Title/Abstract] OR "attitude"[Title/Abstract] OR "attitudes"[Title/Abstract] OR "barrier"[Title/Abstract] OR "barriers"[Title/Abstract] OR "facilitator"[Title/Abstract] OR "facilitators"[Title/Abstract] OR "intent*"[Title/Abstract] | 844,803 |
| 9 | hesitan*[Title/Abstract] OR acceptance[Title/Abstract] OR preference[Title/Abstract] OR rejection[Title/Abstract] OR anti-vaccin*[Title/Abstract] OR attitude[Title/Abstract] OR attitudes[Title/Abstract] OR barrier[Title/Abstract] OR barriers[Title/Abstract] OR facilitator[Title/Abstract] OR facilitators[Title/Abstract] OR intent*[Title/Abstract] | "hesitan*"[Title/Abstract] OR "acceptance"[Title/Abstract] OR "preference"[Title/Abstract] OR "rejection"[Title/Abstract] OR "anti vaccin*"[Title/Abstract] OR "attitude"[Title/Abstract] OR "attitudes"[Title/Abstract] OR "barrier"[Title/Abstract] OR "barriers"[Title/Abstract] OR "facilitator"[Title/Abstract] OR "facilitators"[Title/Abstract] OR "intent*"[Title/Abstract] | 840,497 |
| 8 | ((("vaccination refusal"[MeSH:NoExp]) OR ("anti vaccination movement"[MeSH:NoExp])) OR ("mass vaccination"[MeSH:NoExp])) OR ("vaccination coverage"[MeSH:NoExp]) | "vaccination refusal"[MeSH Terms:noexp] OR "anti vaccination movement"[MeSH Terms:noexp] OR "mass vaccination"[MeSH Terms:noexp] OR "vaccination coverage"[MeSH Terms:noexp] | 5,090 |
| 7 | #3 AND #6 | ("vaccines"[MeSH Terms:noexp] OR ("vaccine"[Title/Abstract] OR "vaccines"[Title/Abstract] OR "vaccination"[Title/Abstract] OR "vaccinations"[Title/Abstract])) AND ("COVID-19"[MeSH Terms:noexp] OR ("COVID-19"[Title/Abstract] OR "ncov*"[Title/Abstract] OR "2019nCoV"[Title/Abstract] OR "19nCoV"[Title/Abstract] OR "covid19*"[Title/Abstract] OR "COVID"[Title/Abstract] OR "SARS-COV-2"[Title/Abstract] OR "SARSCOV-2"[Title/Abstract] OR "SARSCOV2"[Title/Abstract] OR "Severe Acute Respiratory Syndrome Coronavirus 2"[Title/Abstract] OR "Severe Acute Respiratory Syndrome Corona Virus 2"[Title/Abstract])) | 8,326 |
| 6 | #4 OR #5 | "COVID-19"[MeSH Terms:noexp] OR "COVID-19"[Title/Abstract] OR "ncov*"[Title/Abstract] OR "2019nCoV"[Title/Abstract] OR "19nCoV"[Title/Abstract] OR "covid19*"[Title/Abstract] OR "COVID"[Title/Abstract] OR "SARS-COV-2"[Title/Abstract] OR "SARSCOV-2"[Title/Abstract] OR "SARSCOV2"[Title/Abstract] OR "Severe Acute Respiratory Syndrome Coronavirus 2"[Title/Abstract] OR "Severe Acute Respiratory Syndrome Corona Virus 2"[Title/Abstract] | 120,733 |
| 5 | COVID-19[Title/Abstract] OR nCoV*[Title/Abstract] OR 2019nCoV[Title/Abstract] OR 19nCoV[Title/Abstract] OR COVID19*[Title/Abstract] OR COVID[Title/Abstract] OR SARS-COV-2[Title/Abstract] OR SARSCOV-2[Title/Abstract] OR SARSCOV2[Title/Abstract] OR "Severe Acute Respiratory Syndrome Coronavirus 2"[Title/Abstract] OR "Severe Acute Respiratory Syndrome Corona Virus 2"[Title/Abstract] | "COVID-19"[Title/Abstract] OR "ncov*"[Title/Abstract] OR "2019nCoV"[Title/Abstract] OR "19nCoV"[Title/Abstract] OR "covid19*"[Title/Abstract] OR "COVID"[Title/Abstract] OR "SARS-COV-2"[Title/Abstract] OR "SARSCOV-2"[Title/Abstract] OR "SARSCOV2"[Title/Abstract] OR "Severe Acute Respiratory Syndrome Coronavirus 2"[Title/Abstract] OR "Severe Acute Respiratory Syndrome Corona Virus 2"[Title/Abstract] | 116,431 |
| 4 | "covid 19"[MeSH:NoExp] | "covid 19"[MeSH Terms:noexp] | 70,073 |
| 3 | #1 OR #2 | "vaccines"[MeSH Terms:noexp] OR "vaccine"[Title/Abstract] OR "vaccines"[Title/Abstract] OR "vaccination"[Title/Abstract] OR "vaccinations"[Title/Abstract] | 318,648 |
| 2 | vaccine[Title/Abstract] OR vaccines[Title/Abstract] OR vaccination[Title/Abstract] OR vaccinations[Title/Abstract] | "vaccine"[Title/Abstract] OR "vaccines"[Title/Abstract] OR "vaccination"[Title/Abstract] OR "vaccinations"[Title/Abstract] | 315,172 |
| 1 | "vaccines"[MeSH:NoExp] | "vaccines"[MeSH Terms:noexp] | 22,370 |

**Full search strategy for Medline:**

1 Vaccines/ (22367)

2 (vaccine? or vaccination?).ab,ti. (308723)

3 1 or 2 (313284)

4 COVID-19/ (70075)

5 (COVID-19 or nCoV* or 2019nCoV or 19nCoV or COVID19* or COVID or SARS-COV-2 or SARSCOV-2 or SARSCOV2 or

"Severe Acute Respiratory Syndrome Coronavirus 2" or "Severe Acute Respiratory Syndrome Corona Virus 2").ab,ti. (116024)

6 4 or 5 (121281)

7 3 and 6 (8210)

8 Vaccination Refusal/ or Anti-Vaccination Movement/ or Mass Vaccination/ or Vaccination Coverage/ (5092)

9 (hesitan* or acceptance or preference or rejection or anti-vaccin* or attitude? or barrier? or facilitator? or intent*).ab,ti. (835505)

10 8 or 9 (839850)

11 7 and 10 (759)

**Full search strategy for Embase:**

1 vaccine/ (68495)

2 (vaccine? or vaccination?).ab,ti. (360622)

3 1 or 2 (371675)

4 coronavirus disease 2019/ (97101)

5 (COVID-19 or nCoV* or 2019nCoV or 19nCoV or COVID19* or COVID or SARS-COV-2 or SARSCOV-2 or SARSCOV2 or

"Severe Acute Respiratory Syndrome Coronavirus 2" or "Severe Acute Respiratory Syndrome Corona Virus 2").ab,ti. (111782)

6 4 or 5 (118839)

7 3 and 6 (7446)

8 vaccination refusal/ or anti-vaccination movement/ or mass immunization/ or vaccination coverage/ (6789)

9 (hesitan* or acceptance or preference or rejection or anti-vaccin* or attitude? or barrier? or facilitator? or intent*).ab,ti. (1096730)

10 8 or 9 (1102561)

11 7 and 10 (720)

**Full search strategy for PsychInfo:**

| **#** | **Query** | **Limiters/Expanders** | **Last Run Via** | **Results** | |
| --- | --- | --- | --- | --- | --- |
| S9 | S7 AND S8 | Expanders  - Apply equivalent subjects  Search modes  - Find all my search terms | Interface  - EBSCOhost Research Databases  Search Screen  - Advanced Search  Database  - APA PsycInfo | 44 |  |
| S8 | TI ( hesitan* or acceptance or preference or rejection or anti-vaccin*or attitude# or barrier# or facilitator# or intent* ) ORAB ( hesitan* or acceptance or preference or rejection or anti-vaccin*or attitude# or barrier# or facilitator# or intent* ) | Expanders  - Apply equivalent subjects  Search modes  - Find all my search terms | Interface  - EBSCOhost Research Databases  Search Screen  - Advanced Search  Database  - APA PsycInfo | 511,740 |  |
| S7 | S3 AND S6 | Expanders  - Apply equivalent subjects  Search modes  - Find all my search terms | Interface  - EBSCOhost Research Databases  Search Screen  - Advanced Search  Database  - APA PsycInfo | 124 |  |
| S6 | S4 OR S5 | Expanders  - Apply equivalent subjects Search modes  - Find all my search terms | Interface  - EBSCOhost Research Databases Search Screen  - Advanced Search  Database - APA PsycInfo | 5,079 |  |

| S5 | TI ( COVID-19 or nCoV*or 2019nCoV or 19nCoVor COVID19* or COVID orSARS-COV-2 orSARSCOV-2 orSARSCOV2 or "Severe Acute Respiratory Syndrome Coronavirus 2"or "Severe Acute Respiratory Syndrome Corona Virus 2" ) OR AB (COVID-19 or nCoV* or2019nCoV or 19nCoV orCOVID19* or COVID orSARS-COV-2 orSARSCOV-2 orSARSCOV2 or "Severe Acute Respiratory Syndrome Coronavirus 2"or "Severe Acute Respiratory Syndrome Corona Virus 2" ) | Expanders  - Apply equivalent subjects  Search modes  - Find all my search terms | Interface  - EBSCOhost Research Databases  Search Screen  - Advanced Search  Database  - APA PsycInfo | 5,047 |
| --- | --- | --- | --- | --- |
| S4 | DE "COVID-19" | Expanders  - Apply equivalent subjects  Search modes  - Find all my search terms | Interface  - EBSCOhost Research Databases  Search Screen  - Advanced Search  Database  - APA PsycInfo | 1,174 |
| S3 | S1 OR S2 | Expanders  - Apply equivalent subjects  Search modes  - Find all my search terms | Interface  - EBSCOhost Research Databases  Search Screen  - Advanced Search  Database  - APA PsycInfo | 7,689 |
| S2 | TI ( vaccine# OR vaccination# ) OR AB (vaccine# OR vaccination#) | Expanders  - Apply equivalent subjects  Search modes  - Find all my search terms | Interface  - EBSCOhost Research Databases  Search Screen  - Advanced Search  Database  - APA PsycInfo | 6,780 |
| S1 | DE "Immunization" | Expanders  - Apply equivalent subjects   \| Search modes  - Find all my search terms \| \| --- \| | Interface  - EBSCOhost Research Databases  Search Screen  - Advanced   \| Search  Database  - APA PsycInfo \| \| --- \| | 5,049 |

**Full search strategy for CINAHL:**

| # | **Query** | | **Limiters/Expanders** | **Last Run Via** | | **Results** | |  |
| --- | --- | --- | --- | --- | --- | --- | --- | --- |
| S11 | S7 AND S10 | | Expanders  - Apply equivalent subjects  Search modes  - Find allmy search terms | Interface  - EBSCOhost Research Databases  Search Screen  - Advanced Search  Database  - CINAHL Complete | | 182 | |  |
| S10 | S8 OR S9 | | Expanders  - Apply equivalent subjects  Search modes  - Find all my search terms | Interface  - EBSCOhost Research Databases  Search Screen  - Advanced Search  Database  - CINAHL Complete | | 281,269 | |  |
| S9 | TI ( hesitan* or acceptance or preference or rejection or anti-  vaccin*or attitude#  or barrier# or facilitator#  or intent* ) ORAB  ( hesitan* or acceptance  or preference or  rejection or anti-  vaccin*or attitude#  or barrier# or facilitator#  or intent* ) | | Expanders  - Apply equivalent subjects Search modes  - Find all my search terms | Interface  - EBSCOhost Research Databases Search Screen  - Advanced Search  Database  - CINAHL Complete | | 280,736 | |  |
| S8 | | (MH "Anti-Vaccination Movement") OR (MH "Vaccination Coverage") | Expanders  - Apply equivalent subjects  Search modes  - Find all my search terms | | Interface  - EBSCOhost Research Databases  Search Screen  - Advanced Search  Database  - CINAHL Complete | | 664 | |
| S7 | | S3 AND S6 | Expanders  - Apply equivalent subjects  Search modes  - Find all my search terms | | Interface  - EBSCOhost Research Databases  Search Screen  - Advanced Search  Database  - CINAHL Complete | | 1,800 | |
| S6 | | S4 OR S5 | Expanders  - Apply equivalent subjects  Search modes  - Find all my search terms | | Interface  - EBSCOhost Research Databases  Search Screen  - Advanced Search  Database  - CINAHL Complete | | 42,241 | |
| S5 | TI ( COVID-19 or nCoV* or 2019nCoV or 19nCoVor COVID19* or COVID orSARS-COV-2 orSARSCOV-2 orSARSCOV2 or "Severe Acute Respiratory Syndrome Coronavirus 2"or "Severe Acute Respiratory Syndrome Corona Virus 2" ) OR AB (COVID-19 or nCoV* or2019nCoV or 19nCoV orCOVID19* or COVID orSARS-COV-2 orSARSCOV-2 orSARSCOV2 or "Severe Acute Respiratory Syndrome Coronavirus 2"or "Severe Acute Respiratory Syndrome Corona Virus 2" ) | | Expanders  - Apply equivalent  subjects  Search modes  - Find all my search  terms | | Interface  - EBSCOhost Research Databases  Search Screen  - Advanced Search  Database  - CINAHL Complete | |  | |

|  | | | | |
| --- | --- | --- | --- | --- |
| S4 | (MH "COVID-19") | Expanders  - Apply equivalent subjects  Search modes  - Find all my search terms | Interface  - EBSCOhost Research Databases  Search Screen  - Advanced Search  Database  - CINAHL Complete | 14,728 |
| S3 | S1 OR S2 | Expanders  - Apply equivalent subjects  Search modes  - Find all my search terms | Interface  - EBSCOhost Research Databases  Search Screen  - Advanced Search  Database  - CINAHL Complete | 54,538 |
| S2 | TI ( vaccine# OR vaccination# ) OR AB (vaccine# OR vaccination#) | Expanders  - Apply equivalent subjects  Search modes  - Find all my search terms | Interface  - EBSCOhost Research Databases  Search Screen  - Advanced Search  Database  - CINAHL Complete | 51,693 |
| S1 | (MH "Vaccines") | Expanders  - Apply equivalent subjects  Search modes  - Find all my search terms | Interface  - EBSCOhost Research Databases  Search Screen  - Advanced Search  Database  - CINAHL Complete | 9,032 |
