# Supplementary material for: A social ecological approach to identify the barriers and facilitators to COVID-19 vaccination acceptance: A scoping review
Source: PLoS One. 2022 Oct 3;17(10):e0272642. doi: 10.1371/journal.pone.0272642 (PMC9529136; doi:10.1371/journal.pone.0272642)
Supplement: S2 Table — (DOCX) [file pone.0272642.s002.docx]

S2 Table: Details of Study Characteristics

| Author, year | Title | Study Design | Data Collection Methods | Sample | Country |
| --- | --- | --- | --- | --- | --- |
| Gerussi et al., 2021 | Vaccine hesitancy among Italian patients recovered from covid-19 infection towards influenza and sars-cov-2 vaccination | Cross-Sectional | Interview-telephone | 599 | Italy |
| Eguia et al., 2021 | Spain's hesitation at the gates of a covid-19 vaccine | Cross-Sectional | Survey | 731 | Spain |
| Freeman et al., 2020 | COVID-19 Vaccine Hesitancy in the UK: The Oxford Coronavirus Explanations, Attitudes, and Narratives Survey (OCEANS) II | Cross-Sectional | Survey | 5114 | UK |
| Gramacho & Turgeon, 2021 | When politics collides with public health: COVID-19 vaccine country of origin and vaccination acceptance in Brazil | Experiment | Survey | 2771 | Brazil |
| Daly & Robinson, 2021 | Willingness to Vaccinate Against COVID-19 in the U.S.: Representative Longitudinal Evidence From April to October 2020 | Longitudinal | Survey | 7,547 | USA |
| Latkin et al., 2021 | Trust in a COVID-19 vaccine in the US: A social-ecological perspective | Longitudinal | Survey | 592 | USA |
| Tran et al., 2021 | Determinants of COVID-19 vaccine acceptance in a high infection-rate country: a cross-sectional study in Russia | Cross-Sectional | Survey | 876 | Russia |
| Radic et al., 2021 | Intention to take covid-19 vaccine as a precondition for international travel: Application of extended norm-activation model | Cross-Sectional | Survey | 1221 | Multi-countries: South East Asia, China, Africa, South Asia, North America, Europe, Central/South America, Australia/New Zealand |
| Nguyen et al., 2021 | COVID-19 vaccination intent, perceptions, and reasons for not vaccinating among groups prioritized for early vaccination - United States, September and December 2020 | longitudinal | Survey | Multiple samples | USA |
| Kaplan & Milstein, 2021 | Influence of a COVID-19 vaccine's effectiveness and safety profile on vaccination acceptance | Experiment | Survey | 1000 | USA |
| Wang et al., 2021 | The changing acceptance of COVID-19 vaccination in different epidemic phases in China: A longitudinal study | Others: multi-methods | Survey | Multiple samples | China |
| Robertson et al., 2021 | Predictors of COVID-19 vaccine hesitancy in the UK household longitudinal study | Cross-Sectional | Survey | 12,035 | UK |
| Soares et al., 2021 | Factors associated with COVID-19 vaccine hesitancy | Cross-Sectional | Survey | 1943 | Portugal |
| Lueck & Spiers, 2021 | Which Beliefs Predict Intention to Get Vaccinated against COVID-19? A Mixed-Methods Reasoned Action Approach Applied to Health Communication | Other: multi-methods | Survey | Multiple samples | USA |
| Batty et al., 2021 | Pre-pandemic Cognitive Function and COVID-19 Vaccine Hesitancy: Cohort Study | Cohort | Survey | 11,955 | UK |
| Jackson et al., 2021 | Negative vaccine attitudes and intentions to vaccinate against Covid-19 in relation to smoking status: a population survey of UK adults | longitudinal | Survey | 29,148 | UK |
| Yin et al., 2021 | Unfolding the Determinants of COVID-19 Vaccine Acceptance in China | Other: Social Media | Use of public Data (Weibo) | NA | China |
| Hussain et al., 2021 | Artificial intelligence-enabled analysis of UK and US public attitudes on Facebook and Twitter towards COVID-19 vaccinations | Other: Social Media | Use of public data (Facebook and Twitter) | NA | Multi-countries: USA, UK |
| Praveen et al., 2021 | Analyzing the attitude of Indian citizens towards COVID-19 vaccine - A text analytics study | Other: social media (Tweeter) | Use of public Data (Twitter) | NA | India |
| Malesza & Wittmann, 2021 | Acceptance and intake of covid-19 vaccines among older Germans | Cross-Sectional | Survey | 1037 | Germany |
| Kumari et al., 2021 | Knowledge, barriers and facilitators regarding COVID-19 vaccine and vaccination programme among the general population: A cross-sectional survey from one thousand two hundred and forty-nine participants | Cross-Sectional | Survey | 1294 | India |
| Machida et al., 2021 | Acceptance of a covid-19 vaccine in japan during the covid-19 pandemic | Cross-Sectional | Survey | 2956 | Japan |
| Biasio et al., 2021 | Italian adults' likelihood of getting covid-19 vaccine: A second online survey | Cross-Sectional | Survey | 160 | Italy |
| Paul et al., 2020 | Attitudes towards vaccines and intention to vaccinate against COVID-19: Implications for public health communications | Cross-Sectional | Survey | 23,164 | UK |
| Sallam et al., 2021 | High rates of covid-19 vaccine hesitancy and its association with conspiracy beliefs: A study in Jordan and Kuwait among other Arab countries | Cross-Sectional | Survey | 3414 | Multi-countries: Jordan, Kuwait, Saudi Arabia, others |
| Zampetakis & Melas, 2021 | The health belief model predicts vaccination intentions against COVID-19: A survey experiment approach | Experiment | Survey | 1006 | Greece |
| Meier et al., 2021 | Predictors of the intention to receive a SARS-CoV-2 vaccine | Cross-Sectional | Survey | 1054 | USA |
| Petravic et al., 2021 | Factors affecting attitudes towards covid-19 vaccination: An online survey in Slovenia | Cross-Sectional | Survey | 12042 | Slovenia |
| Al-Qerem & Jarab, 2021 | COVID-19 Vaccination Acceptance and Its Associated Factors Among a Middle Eastern Population | Cross-Sectional | Survey | 1,144 | Jordan |
| Alfageeh et al., 2021 | Acceptability of a covid-19 vaccine among the Saudi population | Cross-Sectional | Survey | 2137 | Saudi Arabia |
| Alabdulla et al., 2021 | COVID-19 vaccine hesitancy and attitudes in Qatar: A national cross-sectional survey of a migrant-majority population | Cross-Sectional | Survey | 7821 | Qatar |
| Cordina et al., 2021 | Attitudes towards COVID-19 vaccination, vaccine hesitancy and intention to take the vaccine | Cross-Sectional | Survey | Multiple samples | Multi-countries: Malta, International |
| Benis et al., 2021 | Reasons for taking the COVID-19 vaccine by US social media users | Cross-Sectional | Survey | 1,644 | USA |
| Mercadante & Law 2020 | Will they, or Won't they? Examining patients' vaccine intention for flu and COVID-19 using the Health Belief Model | Cross-Sectional | Survey | 525 | USA |
| Dorman et al., 2021 | Factors Associated with Willingness to be Vaccinated Against COVID-19 in a Large Convenience Sample | Cross-Sectional | Survey | 26,324 | USA |
| Salmon et al., 2021 | COVID-19 vaccination attitudes, values and intentions among United States adults prior to emergency use authorization | Cross-Sectional | Survey | 2,525 | USA |
| Craig 2021 | United States COVID-19 Vaccination Preferences (CVP): 2020 Hindsight | Discrete choice experiment | Survey | 1,153 | USA |
| Allington et al., 2021 | Coronavirus conspiracy suspicions, general vaccine attitudes, trust, and coronavirus information source as predictors of vaccine hesitancy among UK residents during the COVID-19 pandemic | Cross-Sectional | Survey | 4343 | UK |
| Green et al., 2021 | A study of ethnic, gender and educational differences in attitudes toward COVID-19 vaccines in Israel - implications for vaccination implementation policies | Cross-Sectional | Survey | 957 | Israel |
| Griffith et al., 2021 | COVID-19 vaccine hesitancy in Canada: a content analysis of Tweets using the Theoretical Domains Framework | Other: social media | Use of Public Data (Tweets) | NA | Canada |
| Yoda & Katsuyam, 2021 | Willingness to receive covid-19 vaccination in Japan | Cross-Sectional | Survey | 1100 | Japan |
| Chu & Liu, 2021 | Integrating health behavior theories to predict American's intention to receive a COVID-19 vaccine | Cross-Sectional | Survey | 934 | USA |
| Yu et al., 2021 | Understanding the Prevalence and Associated Factors of Behavioral Intention of COVID-19 Vaccination Under Specific Scenarios Combining Effectiveness, Safety, and Cost in the Hong Kong Chinese General Population | Cross-Sectional | Survey | 450 | Hong Kong |
| Liu et al., 2021 | Factors associated with the willingness and acceptance of SARS-CoV-2 vaccine from adult subjects in China | Cross-Sectional | Survey | 983 | China |
| La Vecchia et al., 2020 | Attitudes towards influenza vaccine and a potential COVID-19 vaccine in Italy and differences across occupational groups, September 2020 | Cross-Sectional | Survey | 1,055 | Italy |
| Alqudeimat et al., 2021 | Acceptance of a COVID-19 Vaccine and its Related Determinants among the General Adult Population in Kuwait | Cross-Sectional | Survey | 2,368 | Kuwait |
| McPhedran & Toombs, 2021 | Efficacy or delivery? An online Discrete Choice Experiment to explore preferences for COVID-19 vaccines in the UK | Ddiscrete choice experiment | Survey | 1501 | UK |
| Benham et al., 2021 | Attitudes, current behaviours and barriers to public health measures that reduce COVID-19 transmission: A qualitative study to inform public health messaging | Qualitative | Focus Group | 50 | Canada |
| Loomba et al., 2021 | Measuring the impact of COVID-19 vaccine misinformation on vaccination intent in the UK and USA | Experiment | Survey | 8001 | USA, UK |
| Bokemper et al., 2021 | Timing of COVID-19 vaccine approval and endorsement by public figures | Experiment | Survey | 5,014 | USA |
| Ditekemena et al., 2021 | Covid-19 vaccine acceptance in the democratic republic of congo: A cross-sectional survey | Cross-Sectional | Survey | 4,131 | Congo |
