# Supplementary material for: A social ecological approach to identify the barriers and facilitators to COVID-19 vaccination acceptance: A scoping review
Source: PLoS One. 2022 Oct 3;17(10):e0272642. doi: 10.1371/journal.pone.0272642 (PMC9529136; doi:10.1371/journal.pone.0272642)
Supplement: S4 Table — (DOCX) [file pone.0272642.s004.docx]

Table: Full text screening excluded studies with reasons

| Title | Author, Year | Notes |
| --- | --- | --- |
| Understanding COVID-19 vaccine demand and hesitancy: A nationwide online survey in China | Lin et al., 2020 | Survey completed before Sept 2020/no dates indicated |
| Willingness of the general population to accept and pay for COVID-19 vaccination during the early stages of COVID-19 pandemic: a nationally representative survey in mainland China | Zhang et al., 2021 | Survey completed before Sept 2020/no dates indicated |
| A global survey of potential acceptance of a COVID-19 vaccine | Lazarus et al., 2021 | Survey completed before Sept 2020/no dates indicated |
| Mask usage, social distancing, racial, and gender correlates of COVID-19 vaccine intentions among adults in the US | Latkin et al., 2021 | Survey completed before Sept 2020/no dates indicated |
| Can a COVID-19 vaccine live up to Americans' expectations? A conjoint analysis of how vaccine characteristics influence vaccination intentions | Motta et al., 2021 | Survey completed before Sept 2020/no dates indicated |
| The use of the health belief model to assess predictors of intent to receive the COVID-19 vaccine and willingness to pay | Wong et al., 2020 | Survey completed before Sept 2020/no dates indicated |
| Towards intervention development to increase the uptake of COVID-19 vaccination among those at high risk: Outlining evidence-based and theoretically informed future intervention content | Williams et al., 2020 | Survey completed before Sept 2020/no dates indicated |
| A Proactive Approach for Managing COVID-19: The Importance of Understanding the Motivational Roots of Vaccination Hesitancy for SARS-CoV2 | Taylor et al., 2020 | Survey completed before Sept 2020/no dates indicated |
| A little shot of humility: Intellectual humility predicts vaccination attitudes and intention to vaccinate against COVID-19 | Huynh et al., 2021 | Survey completed before Sept 2020/no dates indicated |
| Fearing the disease or the vaccine: The case of COVID-19 | Karlsson et al., 2021 | Survey completed before Sept 2020/no dates indicated |
| Willingness to get the COVID-19 vaccine with and without emergency use authorization | Guidry et al., 2021 | Survey completed before Sept 2020/no dates indicated |
| Assessing COVID-19 vaccine literacy: a preliminary online survey | Pecorelli et al., 2020 | Survey completed before Sept 2020/no dates indicated |
| Individual preferences for COVID-19 vaccination in China | Leng et al., 2021 | Survey completed before Sept 2020/no dates indicated |
| Predictors of willingness to get a COVID-19 vaccine in the U.S | Kelly et al., 2021 | Survey completed before Sept 2020/no dates indicated |
| Relationship between citizens' health engagement and intention to take the covid-19 vaccine in italy: A mediation analysis | Graffigna et al., 2020 | Survey completed before Sept 2020/no dates indicated |
| Understanding Drivers of COVID-19 Vaccine Hesitancy Among Blacks | Nkwihoreze et al., 2021 | Survey completed before Sept 2020/no dates indicated |
| Intention to Vaccinate Against the Novel 2019 Coronavirus Disease: The Role of Health Locus of Control and Religiosity | Olagoke et al., 2021 | Survey completed before Sept 2020/no dates indicated |
| Psychological characteristics associated with COVID-19 vaccine hesitancy and resistance in Ireland and the United Kingdom | Murphy et al., 2021 | Survey completed before Sept 2020/no dates indicated |
| Acceptance of a Covid-19 vaccine is associated with ability to detect fake news and health literacy | Montagni et al., 2021 | Survey completed before Sept 2020/no dates indicated |
| Hesitant or Not? The Association of Age, Gender, and Education with Potential Acceptance of a COVID-19 Vaccine: A Country-level Analysis | Lazarus et al., 2020 | Survey completed before Sept 2020/no dates indicated |
| Willingness of Greek general population to get a COVID-19 vaccine | Kourlaba et al., 2021 | Survey completed before Sept 2020/no dates indicated |
| Acceptance of a COVID-19 Vaccine in Southeast Asia: A Cross-Sectional Study in Indonesia | Harapan et al., 2020 | Survey completed before Sept 2020/no dates indicated |
| Influences on attitudes regarding potential covid-19 vaccination in the united states | Pogue et al., 2020D30 | Survey completed before Sept 2020/no dates indicated |
| What Indians Think of the COVID-19 vaccine: A qualitative study comprising focus group discussions and thematic analysis | Kumari et al., 2021 | Survey completed before Sept 2020/no dates indicated |
| Quantifying the Impact of Public Perceptions on Vaccine Acceptance Using Behavioral Economics | Hursh et al., 2020 | Survey completed before Sept 2020/no dates indicated |
| Coronavirus conspiracy beliefs, mistrust, and compliance with government guidelines in England | Freeman et al., 2020 | Survey completed before Sept 2020/no dates indicated |
| Acceptance of covid-19 vaccination during the covid-19 pandemic in china | Wang et al., 2020 | Survey completed before Sept 2020/no dates indicated |
| COVID-19 vaccine hesitancy in a representative working-age population in France: a survey experiment based on vaccine characteristics | Schwarzinger et al., 2021 | Survey completed before Sept 2020/no dates indicated |
| The French public's attitudes to a future COVID-19 vaccine: The politicization of a public health issue | Ward et al., 2020 | Survey completed before Sept 2020/no dates indicated |
| The Persistence of Vaccine Hesitancy: COVID-19 Vaccination Intention in New Zealand | Thaker, 2021 | Survey completed before Sept 2020/no dates indicated |
| COVID-19 vaccination intention in the UK: results from the COVID-19 vaccination acceptability study (CoVAccS), a nationally representative cross-sectional survey | Sherman et al., 2020 | Survey completed before Sept 2020/no dates indicated |
| Susceptibility to misinformation about COVID-19 around the world | Roozenbeek et al., 2020 | Survey completed before Sept 2020/no dates indicated |
| Predicting Intentions to Vaccinate against COVID-19 and Seasonal Flu: The Role of Consideration of Future and Immediate Consequences | Ma and Ma, 2021 | Survey completed before Sept 2020/no dates indicated |
| Factors influencing likelihood of COVID-19 vaccination: A survey of Tennessee adults | Gatwood et al., 2021 | Survey completed before Sept 2020/no dates indicated |
| Public Perceptions of COVID-19 in Australia: Perceived Risk, Knowledge, Health-Protective Behaviors, and Vaccine Intentions | Faasse and Newby, 2020 | Survey completed before Sept 2020/no dates indicated |
| COVID-19 conspiracy beliefs, health behaviors, and policy support | Earnshaw et al., 2020 | Survey completed before Sept 2020/no dates indicated |
| Intention to participate in a COVID-19 vaccine clinical trial and to get vaccinated against COVID-19 in France during the pandemic | Detoc et al., 2020 | Survey completed before Sept 2020/no dates indicated |
| Correlates and disparities of intention to vaccinate against COVID-19 | Callaghan et al., 2021 | Survey completed before Sept 2020/no dates indicated |
| Determinants of covid-19 vaccine acceptance in saudi arabia: A web-based national survey | Al-Mohaithef et al., 2020 | Survey completed before Sept 2020/no dates indicated |
| Flattening the curve of covid-19 vaccine rejection-an international overview | Feleszko et al., 2021 | Survey completed before Sept 2020/no dates indicated |
| Social media exposure, risk perception, preventive behaviors and attitudes during the COVID-19 epidemic in la Paz, Bolivia: A cross sectional study | Fernandez et al., 2021 | Survey completed before Sept 2020/no dates indicated |
| COVID-19 vaccine hesitancy and resistance: Correlates in a nationally representative longitudinal survey of the Australian population | Edwards et al., 2021 | Survey completed before Sept 2020/no dates indicated |
| Attitudes Toward a Potential SARS-CoV-2 Vaccine : A Survey of U.S. Adults | Fisher et al., 2020 | Survey completed before Sept 2020/no dates indicated |
| Associations of COVID-19 risk perception with vaccine hesitancy over time for Italian residents | Caserotti et al., 2021 | Survey completed before Sept 2020/no dates indicated |
| Public preference for COVID‐19 vaccines in China: A discrete choice experiment | Dong et al., 2020 | Survey completed before Sept 2020/no dates indicated |
| Assessment of vaccine hesitancy to a covid-19 vaccine in cameroonian adults and its global implication | Dinga et al., 2021 | Survey completed before Sept 2020/no dates indicated |
| Factors associated with the intention to obtain a COVID-19 vaccine among a racially/ethnically diverse sample of women in the USA | Allen et al., 2021 | Survey completed before Sept 2020/no dates indicated |
| How scientific reasoning correlates with health-related beliefs and behaviors during the COVID-19 pandemic? | Cavojova et al., 2020 | Survey completed before Sept 2020/no dates indicated |
| Converting the maybes: Crucial for a successful COVID-19 vaccination strategy | Attwell et al., 2021 | Survey completed before Sept 2020/no dates indicated |
| COVID-19 vaccine intentions in the United States, a social-ecological framework | Dayton et al., 2021 | Survey completed before Sept 2020/no dates indicated |
| Analyzing Attitude towards COVID-19 Vaccine in the Context of the Health Industry: The Role of Country of Origin Image | Aydin et al., 2021 | Survey completed before Sept 2020/no dates indicated |
| An online survey of the attitude and willingness of Chinese adults to receive COVID-19 vaccination | Chen et al., 2021 | Survey completed before Sept 2020/no dates indicated |
| Conspiracy Beliefs, Rejection of Vaccination, and Support for hydroxychloroquine: A Conceptual Replication-Extension in the COVID-19 Pandemic Context | Bertin et al., 2020 | Survey completed before Sept 2020/no dates indicated |
| As the Pandemic Progresses, How Does Willingness to Vaccinate against COVID-19 Evolve? | Alley et al., 2021 | Survey completed before Sept 2020/no dates indicated |
| Vaccine hesitancy: the next challenge in the fight against COVID-19 | Dror et al., 2020 | Survey completed before Sept 2020/no dates indicated |
| Do Messages Matter? Investigating the Combined Effects of Framing, Outcome Uncertainty, and Number Format on COVID-19 Vaccination Attitudes and Intention | Chen et al., 2021 | Survey completed before Sept 2020/no dates indicated |
| Preferences for a COVID-19 vaccine in Australia | Borriello et al., 2021 | Survey completed before Sept 2020/no dates indicated |
| Media usage predicts intention to be vaccinated against SARS-CoV-2 in the US and the UK | Allington et al., 2021 | Survey completed before Sept 2020/no dates indicated |
| While studies on COVID-19 vaccine is ongoing, the public's thoughts and attitudes to the future COVID-19 vaccine | Akarsu et al., 2021 | Survey completed before Sept 2020/no dates indicated |
| When it is available, will we take it? Social media users' perception of hypothetical covid-19 vaccine in nigeria | Adebisi et al., 2021 | Survey completed before Sept 2020/no dates indicated |
| Unfolding determinants of COVID-19 vaccine acceptance in China | Yin et al., 2021 | Duplicate |
| Once we have it, will we use it? A European survey on willingness to be vaccinated against COVID-19 | Neumann-Böhme et al., 2020 | Wrong study design (e.g. protocol) |
| National trends in the US public’s likelihood of getting a COVID-19 vaccine—April 1 to December 8, 2020 | Szilagyi et al., 2021 | Wrong study design (e.g. protocol) |
| Perceptions, Knowledge, and Behaviors Related to COVID-19 Among Social Media Users: Cross-Sectional Study | Ali et al., 2020 | [No study/data on public's perspectives reported](https://preprints.jmir.org/preprint/19913) |
| Will vaccination refusal prolong the war on SARS-CoV-2? | Marcec et al., 2021 | Wrong study design (e.g. protocol) |
| COVID-19 Vaccine Acceptance: We Need to Start Now | Kuppalli et al., 2021 | Wrong study design (e.g. protocol) |
| Life History Orientation Predicts COVID-19 Precautions and Projected Behaviors | Corpuz et al., 2020 | No study/data on public's perspectives reported |
| Intention to vaccinate against COVID-19 in Australia | Rhodes et al., 2021 | Wrong study design (e.g. protocol) |
| "Cultivating" acceptance of a COVID-19 vaccination program: Lessons from Italy | Graffigna et al., 2020 | Wrong study design (e.g. protocol) |
| Acceptance of a COVID-19 vaccine: A multifactorial consideration | Garcia et al., 2020 | Wrong study design (e.g. protocol) |
| PSU20 Attitude of Hungarian Adults Towards Emergency Plans before COVID-19 Outbreak in Hungary | Kovacs et al., 2020 | No study/data on public's perspectives reported; |
| The power of choice: Experimental evidence that freedom to choose a vaccine against COVID-19 improves willingness to be vaccinated | Sprengholz et al., 2021 | Wrong study design (e.g. protocol) |
| Covid-19: Vaccine hesitancy fell after vaccination programme started | Moberly, 2021 | Wrong study design (e.g. protocol) |
| Looking inside the "black box" of vaccine hesitancy: unlocking the effect of psychological attitudes and beliefs on COVID-19 vaccine acceptance and implications for public health communication | Barello et al., 2021 | Wrong study design (e.g. protocol) |
| A future vaccination campaign against COVID-19 at risk of vaccine hesitancy and politicisation | COCONEL Group, 2020 | Wrong study design (e.g. protocol) |
| A three-tiered approach to address barriers to COVID-19 vaccine delivery in the Black community | Abdul-Mutakabbir et al., 2021 | Wrong study design (e.g. protocol) |
| A COVID-19 vaccine success? patients perception of Covid vaccination program | Wardha et al., 2021 | No study/data on public's perspectives reported |
| The COVID-19 Vaccine Blind Spot: Transparency and open-dialogue are vital to sway the vaccine-hesitant community | Thomas, 2020 | No study/data on public's perspectives reported |
| Covid-19 vaccine hesitancy among ethnic minority groups | Razai et al., 2021 | Wrong study design (e.g. protocol) |
| Fight against hesitancy: public health concern towards COVID-19 vaccine | Punsalan , 2021 | Wrong study design (e.g. protocol) |
| Covid-19: Use social media to maximise vaccine confidence and uptake | Patten et al., 2021 | Wrong study design (e.g. protocol) |
| How to Face the Advent of SARS-CoV-2 Vaccination in IBD Patients: Another Task for Gastroenterologists | Papa et al., 2021 | Wrong study design (e.g. protocol) |
| No psychological vaccination: Vaccine hesitancy is associated with negative psychiatric outcomes among Israelis who received COVID-19 vaccination | Palgi et al., 2021 | No study/data on public's perspectives reported |
| Even covid-19 can't kill the anti-vaccination movement | Megget, 2020 | Wrong study design (e.g. protocol) |
| Breaking the Spell: Fighting Myths About COVID-19 Vaccination | Khawaja et al., 2021 | Wrong study design (e.g. protocol) |
| Faster than warp speed: early attention to COVD-19 by anti-vaccine groups on Facebook | Kalichman et al., 2021 | No study/data on public's perspectives reported |
| BAME community hesitancy in the UK for COVID-19 vaccine: Suggested solutions | Iyengar et al., 2021 | Wrong study design (e.g. protocol) |
| Correcting COVID-19 vaccine misinformation: Lancet Commission on COVID-19 Vaccines and Therapeutics Task Force Members* | Hotez et al., 2021 | Wrong study design (e.g. protocol) |
| Tackling vaccine hesitancy during the COVID-19 pandemic | Heffernan, 2020 | Wrong study design (e.g. protocol) |
| Anti-SARS-CoV-2 vaccination strategy for pregnant women in Japan | Hayakawa et al., 2021 | No study/data on public's perspectives reported |
| The Scientists' Collective 10-point proposal for equitable and timeous access to COVID-19 vaccine in South Africa | Gray et al., 2020 | Wrong study design (e.g. protocol) |
| COVID-19, fake news, and vaccines: Should regulation be implemented? | Gonzalez-De-Julian et al., 2021 | Wrong study design (e.g. protocol) |
| Tackling sheepishness about a COVID-19 vaccine for the sake of herd immunity | Gildea, 2020 | Wrong study design (e.g. protocol) |
| The COVID-19 and Influenza "Twindemic": Barriers to Influenza Vaccination and Potential Acceptance of SARS-CoV2 Vaccination in African Americans | Ferdinand et al., 2020 | Perspective reported is on other vaccination, not COVID 19 |
| 'It's not the science we distrust; it's the scientists': Reframing the anti-vaccination movement within Black communities | Batelaan, 2021 | Wrong study design (e.g. protocol) |
| First-Dose COVID-19 Vaccination Coverage among Skilled Nursing Facility Residents and Staff | Gharpure et al., 2021 | No study/data on public's perspectives reported ; |
| Barriers to vaccination for coronavirus disease 2019 (COVID-19) control: experience from the United States | Zhang et al., 2021 | Wrong study design (e.g. protocol) |
| Acceptance of COVID-19 vaccination among jordanian adults: a cross sectional study | Salama and Almaaytah, 2020 | Unable to find full text |
| Vaccine Hesitancy and Demand for Immunization in Eastern Europe and Central Asia: Implications for the Region and Beyond | Obregon et al., 2020 | Wrong study design (e.g. protocol) |
| Systemic racism and overcoming my COVID-19 vaccine hesitancy | Nephew, 2021 | No study/data on public's perspectives reported |
| Medical experimentation and the roots of COVID-19 vaccine hesitancy among Indigenous Peoples in Canada | Mosby and Swidrovich, 2021 | No study/data on public's perspectives reported |
| What Is the World Doing about COVID-19 Vaccine Acceptance? | MacPherson, 2020 | Wrong study design (e.g. protocol) |
| Volatility of vaccine confidence | Larson and Broniatowski, 2021 | No study/data on public's perspectives reported |
| What money can't buy: an argument against paying people to get vaccinated | Jecker, 2021 | No study/data on public's perspectives reported |
| In the service of the Filipino: the role of Catholic higher education institutions in promoting COVID-19 vaccines in the Philippines | Vicente and Cordero, 2021 | No study/data on public's perspectives reported |
| Understanding the determinants of acceptance of COVID-19 vaccines: a challenge in a fast-moving situation | Verger and Peretti-Watel, 2021 | No study/data on public's perspectives reported |
| COVID-19 vaccine rollout: will it affect the rates of vaccine hesitancy in Africa? | Ekwebelem et al., 2021 | Wrong study design (e.g. protocol) |
| Employer-Mandated Vaccination for COVID-19 | Rothstein et al., 2021 | No study/data on public's perspectives reported |
| Covid-19 vaccine roll-out in south africa and zimbabwe: Urgent need to address community preparedness, fears and hesitancy | Dzinamarira et al., 2021 | Wrong study design (e.g. protocol) |
| Exploring Why Adult Mexican Males Do Not Get Vaccinated: Implications for COVID-19 Preventive Actions | Snyder et al., 2020 | Perspective reported is on other vaccination, not COVID 19 |
| COVID-19 vaccination for people with severe mental illness: why, what, and how? | Mazereel et al., 2021 | Wrong study design (e.g. protocol) |
| Erratum: Author Correction: Measuring the impact of COVID-19 vaccine misinformation on vaccination intent in the UK and USA (Nature human behaviour (2021) 5 3 (337-348)) | Loomba et al., 2021 | Wrong study design (e.g. protocol) |
| Addressing Justified Vaccine Hesitancy in the Black Community | Laurencin, 2021 | No study/data on public's perspectives reported |
| Willingness to Wait for a Vaccine Against COVID-19: Results of a Preference Survey | Krucien et al., 2020 | No study/data on public's perspectives reported |
| How can a global pandemic affect vaccine hesitancy? | Dube and MacDonald, 2020 | Wrong study design (e.g. protocol) |
| Expert opinion in mental disorder: Why is acceptance of the COVID-19 vaccines so problematic? | Gorman et al., 2021 | No study/data on public's perspectives reported |
| Are we ready for the arrival of the new covid-19 vaccinations? Great promises and unknown challenges still to come | Gori et al., 2021 | No study/data on public's perspectives reported |
| Dispelling anti-vaxxer misinformation about COVID-19 vaccination | Glasper, 2021 | Wrong study design (e.g. protocol) |
| Addressing the elephant in the room: COVID-19 vaccine hesitancy in Black and Asian communities | Darko , 2021 | No study/data on public's perspectives reported |
| What must be done to tackle vaccine hesitancy and barriers to COVID-19 vaccination in migrants? | Crawshaw et al., 2021 | No study/data on public's perspectives reported |
| COVID-19 Vaccination of Adolescents and Young Adults of Color: Viewing Acceptance and Uptake With a Health Equity Lens | Coyne-Beasley et al., 2021 | Wrong study design (e.g. protocol) |
| Multisectoral Approach on COVID-19 vaccination: a proposed solution on vaccine hesitancy | Corpuz, 2021 | No study/data on public's perspectives reported |
| COVID-19 Vaccine Hesitancy: Shortening the Last Mile | Chevallier et al., 2021 | No study/data on public's perspectives reported |
| Efforts towards a COVID-19 vaccine | Brussow, 2020 | No study/data on public's perspectives reported |
| The public's role in COVID-19 vaccination: Human-centered recommendations to enhance pandemic vaccine awareness, access, and acceptance in the United States | Brunson et al., 2020 | No study/data on public's perspectives reported |
| "When will we have a vaccine?" - Understanding questions and answers about covid-19 vaccination | Bloom et al., 2020 | No study/data on public's perspectives reported |
| Barriers to Administering Vaccines in Inflammatory Bowel Disease Centers | Bhat et al., 2021 | No study/data on public's perspectives reported |
| Determinants of COVID-19 vaccine acceptance in the US | Malik et al., 2020 | Survey completed before Sept 2020/no dates indicated |
| COVID-19 Vaccination Hesitancy in the United States: A Rapid National Assessment | Khubchandani et al., 2021 | Survey completed before Sept 2020/no dates indicated |
| To what extent are conspiracy theorists concerned for self versus others? A COVID-19 test case | Hornsey et al., 2021 | Survey completed before Sept 2020/no dates indicated |
| A National Survey Assessing SARS-CoV-2 Vaccination Intentions: Implications for Future Public Health Communication Efforts | Head et al., 2020 | Survey completed before Sept 2020/no dates indicated |
| Intention to receive a vaccine against SARS-CoV-2 in Italy and its association with trust, worry and beliefs about the origin of the virus | Prati, 2020 | Survey completed before Sept 2020/no dates indicated |
| Mistrust in biomedical research and vaccine hesitancy: the forefront challenge in the battle against COVID-19 in Italy | Palamenghi et al., 2020 | Survey completed before Sept 2020/no dates indicated |
| Factors Associated With US Adults' Likelihood of Accepting COVID-19 Vaccination | Kreps et al., 2020 | [Survey completed before Sept 2020/no dates indicated](https://jamanetwork.com/journals/jamanetworkopen/fullarticle/2773314) |
| Examining Australian public perceptions and behaviors towards a future COVID-19 vaccine | Seale et al., 2021 | Survey completed before Sept 2020/no dates indicated |
| Predictors of intention to vaccinate against COVID-19: Results of a nationwide survey | Ruiz et al., 2021 | Survey completed before Sept 2020/no dates indicated |
| Acceptability of a COVID-19 vaccine among adults in the United States: How many people would get vaccinated? | Reiter et al., 2020 | Survey completed before Sept 2020/no dates indicated |
| Acceptance of the COVID-19 vaccine based on the health belief model: A population-based survey in Hong Kong | Wong et al., 2021 | Survey completed before Sept 2020/no dates indicated |
| What drives resistance to Public Health measures in Canada's COVID-19 pandemic? An online survey of Canadians' knowledge, attitudes, and practices | Underschultz et al., 2021 | Survey completed before Sept 2020/no dates indicated |
| Unwillingness to engage in behaviors that protect against COVID-19: the role of conspiracy beliefs, trust, and endorsement of complementary and alternative medicine | Soveri et al., 2021 | Survey completed before Sept 2020/no dates indicated |
| Conspiracy theories as barriers to controlling the spread of COVID-19 in the U.S | Romer et al., 2020 | Survey completed before Sept 2020/no dates indicated |
| An Investigation of Low COVID-19 Vaccination Intentions among Black Americans: The Role of Behavioral Beliefs and Trust in COVID-19 Information Sources | Woko et al., 2020 | Survey completed before Sept 2020/no dates indicated |
| Social patterning and stability of intention to accept a COVID-19 vaccine in scotland: Will those most at risk accept a vaccine? | Williams et al., 2021 | Survey completed before Sept 2020/no dates indicated |
| Improving COVID-19 vaccine acceptance: Including insights from human decision-making under conditions of uncertainty and human-centered design | Poland et al., 2021 | No study/data on public's perspectives reported |
| Officials gird for a war on vaccine misinformation | Cornwall, 2020 | Wrong study design (e.g. protocol) |
| Mindfulness as key in easing COVID-19 vaccine hesitancy | Capulong, 2021 | No study/data on public's perspectives reported |
| Hopes, hesitancy and the risky business of vaccine development | Calnan and Douglas, 2020 | No study/data on public's perspectives reported |
| The race to a COVID-19 vaccine: Opportunities and challenges in development and distribution | Burgos et al., 2021 | No study/data on public's perspectives reported |
| Harnessing the nursing contribution to COVID-19 mass vaccination programmes: Addressing hesitancy and promoting confidence | Burden et al., 2021 | No study/data on public's perspectives reported |
| A Tale of Two Crises: Addressing Covid-19 Vaccine Hesitancy as Promoting Racial Justice | Bunch, 2021 | No study/data on public's perspectives reported |
| Beyond politics: additional factors underlying skepticism of a COVID-19 vaccine | Boyd 2021 | No study/data on public's perspectives reported |
| Politicizing public health: the powder keg of rushing COVID-19 vaccines | Limaye et al., 2020 | No study/data on public's perspectives reported |
| COVID-19 Vaccine: Why the Hesitancy? | Grossman, 2021 | Wrong study design (e.g. protocol) |
| Vaccine hesitancy in low- And middle-income countries: Potential implications for the COVID-19 response | Bhopal and Nielsen, 2021 | Perspective reported is on other vaccination, not COVID 19 |
| COVID-19: vaccination in a developing country | Gutierrez-Zevallos et al., 2021 | Wrong study design (e.g. protocol) |
| Vaccine hesitancy in the University of Malta Faculties of Health Sciences, Dentistry and Medicine vis-a-vis influenza and novel COVID-19 vaccination | Grech and Gauci, 2020 | Unable to find full text; |
| The anti-vaccination infodemic on social media: A behavioral analysis | Germani and Biller-Andromo, 2021 | No study/data on public's perspectives reported |
| Lessons Relearned? H1N1, COVID-19, and Vaccination Planning | Fraser and Blumenstock, 2021 | No study/data on public's perspectives reported |
| Prescriber Hesitancy on COVID Vaccines: Data suggests physicians just as hesitant as patients, but recent successes could turn the tide | Fitzgerald, 2021 | No study/data on public's perspectives reported |
| Commentary: Addressing vaccine hesitancy in the age of COVID-19 | Fisher et al., 2021 | No study/data on public's perspectives reported |
| Evidence-Based Strategies for Clinical Organizations to Address COVID-19 Vaccine Hesitancy | FinneyRutten et al.,2021 | Wrong study design (e.g. protocol) |
| Expert: Anti-Vaxxers Will Try to Undermine Pandemic Vaccine: Group moves to civil rights arguments for refusing immunization | Evans, 2020 | No study/data on public's perspectives reported |
| Anti-vaccine movement could undermine efforts to end coronavirus pandemic, researchers warn | Ball, 2020 | Wrong study design (e.g. protocol) |
| SARS-CoV-2 vaccination for patients with inflammatory bowel disease: a British Society of Gastroenterology Inflammatory Bowel Disease section and IBD Clinical Research Group position statement | Alexander et al., 2021 | No study/data on public's perspectives reported |
| Preparing the Community for a Vaccine Against COVID-19 | AlAwaidy and Khamis, 2020 | No study/data on public's perspectives reported |
| COVID-19 Vaccines for Healthcare Workers: The Good, the Bad, and the Ugly: Many stepping up amid chaos and confusion | Ahc 2021 | No study/data on public's perspectives reported |
| Help Case Managers Overcome Vaccine Hesitancy: Distrust can be nuanced, personal | Ahc 2021 | No study/data on public's perspectives reported |
| Research World Can Help Build Trust Among Minorities: Context helps in understanding | Ahc 2021 | No study/data on public's perspectives reported |
| The Age of Misinformation and Vaccine Hesitancy: Former CDC director: 'Be first, be right, be credible' | Ahc 2020 | No study/data on public's perspectives reported |
| The COVID-19 Vaccine Quarantine: A New Staffing Headache | Ahc 2021 | Unable to find full text |
| Prioritizing COVID-19 vaccinations for individuals with intellectual and developmental disabilities | Hotez et al., 2021 | No study/data on public's perspectives reported |
| Reassuring the Public and Clinical Community About the Scientific Review and Approval of a COVID-19 Vaccine | Bauchner et al., 2020 | No study/data on public's perspectives reported |
| Building public trust in COVID-19 vaccines through the Catholic Church in the Philippines | Gopez, 2021 | No study/data on public's perspectives reported |
| ASHP principles for COVID-19 vaccine distribution, allocation, and mass immunization | Anonymous 2020 | No study/data on public's perspectives reported |
| The COVID vaccine challenges that lie ahead | Anonymous 2020 | No study/data on public's perspectives reported |
| Challenges in creating herd immunity to SARS-CoV-2 infection by mass vaccination | Anderson et al., 2020 | [No study/data on public's perspectives reported](https://doi.org/10.1016/S0140-6736(20)32318-7) |
| Ramadan and COVID-19 vaccine hesitancy-a call for action | Ali et al., 2021 | No study/data on public's perspectives reported |
| How Family Planning Providers Can Handle Challenges of COVID-19 Vaccine Rollout: Issues with logistics, staff hesitancy | Ahc 2021 | No study/data on public's perspectives reported |
| Providers Can Reduce Vaccine Hesitancy Among Staff: Some people are "anti-first," not anti-vax | Ahc 2021 | No study/data on public's perspectives reported |
| Dealing with vaccine hesitancy in Africa: The prospective COVID-19 vaccine context | Afolabi et al., 2021 | No study/data on public's perspectives reported |
| COVID-19 vaccination strategies in public psychiatry | Fetter, 2021 | No study/data on public's perspectives reported |
| COVID-19 Vaccination and the Challenge of Infodemic and Disinformation | Farooq and Rathore 2021 | No study/data on public's perspectives reported |
